# Heading: Efficacy of Lactic Acid Bacteria Isolated from Winemaking Byproducts in Preventing Alcoholic Liver Injury

**DOI:** 10.1007/s12088-025-01506-8

**Published:** 2025-07-16

**Authors:** Min-Wei Hsieh, Huey-Yueh Chen, Cheng-Chih Tsai

**Affiliations:** 1 Taiwan Tobacco and Liquor Corporation, Zhongzheng District, Taipei City, 10066 Taiwan; 2 Department of Food Science and Technology, HungKuang University, No. 1018, Sec. 6, Taiwan Boulevard, Shalu District, Taichung City, 43302 Taiwan

**Keywords:** Alcoholic liver, Probiotics, Inflammation, Glutathione peroxidase, Triglycerides

## Abstract

The primary cause of liver damage due to alcohol consumption is long-term intake that disrupts normal metabolism. We focused on evaluating the screening of lactic acid bacteria (LAB) derived from winemaking byproducts to prevent alcoholic liver damage. We employed in vitro cell tests to determine the effect of probiotics on reducing cytokine levels (INF-*γ*, TNF-*α*, IL-6, IL-8) and alcohol content after LPS-induced inflammation of alcoholic liver cells. We also analyzed the levels of aspartate transaminase (AST) and alanine transaminase (ALT) after hepatocyte injury. The results showed that strain tau 2–8 (107) and strain tau 1–1 (107) could maintain a viable count above 6 Log CFU/mL at 20% alcohol concentration. Moreover, strain 3–14 (106) and strain 6–10 (107) effectively reduced AST content in HepG2 cells stimulated by alcohol, with clearance rates of 107 and 105%, respectively. In the animal experiments, feeding single and combination multi-strains at the eighth week resulted in AST levels decrease significantly compared to the alcohol group in serum. Specifically, the single strain 3–14 (106) and the combined multi-strain had better effects, reducing AST levels by 54 and 48%, respectively. Additionally, the serum triglyceride content of the group under an alcoholic diet increased over time, but feeding with lactic acid bacteria resulted in a significant decrease. The glutathione peroxidase enzyme activity of the alcohol group decreased by 31.9% compared to the control group, while feeding with single-strain lactobacillus and combined lactobacillus groups increased significantly. Feeding lactic acid bacteria effectively reduced and improved alcoholic fatty liver and liver damage. In this study, LAB derived from the utilization of winemaking by-products also helps to manage wine industrial waste in an efficient way.

## Introduction

The liver is an essential organ responsible for performing numerous vital functions in the body, including nutrient metabolism, detoxification, hormone regulation, and bile secretion. Additionally, the liver helps maintain energy balance and plays a significant role in the body’s immune system [1]. AST and ALT are widely recognized enzymes associated with liver function that can serve as biomarkers of liver health [2]. Hepatocyte injury can increase the serum concentrations of these enzymes, which are indicative of hepatic damage [3].

Alcoholic liver disease is primarily caused by long-term alcohol intake, which increases the concentration of toxins in the intestinal tract [4]. This can lead to changes in intestinal epithelial permeability and increased bacterial growth, including gram-negative bacteria, in the intestinal tract [5, 6]. As a result, endotoxins, particularly lipopolysaccharide (LPS), are released, and there is an increased risk of LPS transfer from the intestinal lumen to the hepatic blood flow or lymph. This process induces and exacerbates alcoholic liver disease [7]. Increased LPS concentration can also lead to oxidative stress due to the generation of reactive oxygen species. This process induces macrophage Kupffer cells to produce several cytokines, such as tumor necrosis factor-*α* (TNF-*α*) and interleukin (IL)-1, IL-6, IL-8, and other inflammatory substances that eventually lead to hepatic inflammation [8, 9]. Therefore, maintaining the balance of gut flora is critical for preventing alcoholic liver injury [10, 11].

Cell damage caused by oxidative stress can be prevented by antioxidant defense systems. However, chronic alcohol intake can induce free radical production, leading to oxidative stress that further affects cell integrity [12, 13]. Antioxidant defense systems are classified into enzymatic (e.g., catalase, glutathione reductase, superoxide dismutase and glutathione peroxidase) and non-enzymatic (e.g., vitamin A, vitamin E, glutathione and alpha-lipoic acid) categories. Therefore, GSH and GSH Px activity are commonly used markers for liver function [12–14]. LAB have been used in recent years to alleviate alcoholic liver disease [15, 16]. Feeding C57BL/6N mice with LAB can effectively reduce serum AST and ALT concentrations and decrease triglyceride and cholesterol accumulation in the liver, thus preventing alcoholic liver disease and fatty liver [17]. Probiotics protect against alcoholic liver injury primarily through three mechanisms: (i) inhibiting LPS production, (ii) increasing antioxidant activity to scavenge free radicals, and (iii) mitigating inflammation and strengthening the organism’s immunocompetence [18–21]. In a mouse model, feeding strains of *Lactobacillus plantarum* HFY09 reduced toxin production and mitigated the severity of alcohol-induced liver injury [18]. Additionally, *Lactobacillus* spp. reduces liver oxidative stress and inflammation induced by alcohol [19].

In this study, a screening of 33 LAB strains was performed to investigate their potential hepatoprotective effects in HepG2 cells. Strains that demonstrated promising results in the initial cell-based assay were identified and subjected to small-scale cultivation and trial production. A master formula based on these strains was designed, followed by an animal experiment to evaluate the effect of the probiotic on blood biochemical markers and liver histopathology. To confirm the effectiveness of the probiotic in preventing alcoholic liver injury, GSH concentration, GSH Px activity, changes in liver TG levels, as well as the inflammatory response and cytokine secretion were analyzed. The LAB strains that were selected from the in vitro experiment included *Pediococcus pentosaceus* strain (106) 3–14, *Lactobacillus plantarum* strain (107) tau 6–2, and *L. plantarum* strain (107) 8–16. In the animal experiments, a single strain of LAB and a combination of LAB were used to investigate their efficacy in alleviating or reducing alcoholic fatty liver.

## Materials and Methods

### Strains Growth Conditions

We utilized 33 LAB strains isolated from winemaking byproducts provided by the Taiwan Tobacco and Liquor Corporation (Taipei, Taiwan) [22]. These strains demonstrated specific Probiotics [22] and were suspended in lactobacilli MRS broth (Difco Laboratories, Detroit, MI, USA) (contained 15% glycerol) before being stored at − 80 °C. Prior to experimentation, they were reactivated twice using lactobacilli MRS broth (Difco Laboratories, Detroit, MI, USA) supplemented with 0.05% L-cysteine. The strains were then cultured for 24 h under optimal conditions at 37 °C.

### The Anti-inflammatory Effects of Probiotics

The HepG2 human liver cancer cell and mouse macrophage (RAW 264.7) cell lines were procured from Bioresource Collection and Research Center (Hsinchu City, Taiwan). HepG2 cells were maintained in Eagle’s minimum essential medium (MEM), 10% fetal bovine serum (FBS), 1% nonessential amino acids (NEAA), and 1.5 g sodium bicarbonate. RAW 264.7 cells were maintained in Dulbecco’s modified Eagle’s medium (DMEM), 10% FBS and 1.5 g sodium bicarbonate. HepG2 cells (2 × 10^5^ cells/well) for IL-8 and RAW264.7 cells (1 × 10^5^ cell/well) for IL-6 and TNF-*α* were inoculated into 24-well plates. Upon reaching 80% confluency, the cells were washed once with 1 × phosphate-buffered saline (PBS) and 800 μL of fresh cell culture medium was added. Subsequently, 100 μL of LAB suspension (containing 10^9^ colony-forming units (CFU)/mL) and 100 μL of lipopolysaccharide (LPS) (100 ng/mL) were added. The bacterial suspension was pre-washed twice with 1 × PBS, the supernatant was removed, and the suspension was reconstituted using the cell culture medium. The plates were then incubated in a 5% CO_2_ incubator at 37 °C for 24 h. The cell supernatant was collected and stored at − 80 °C until analysis of IL-8, IL-6, and TNF-*α* levels.

### Alcohol Tolerance Test

One hundred μL of the cultures was inoculated into 4 mL of MRS broth, with or without alcohol, to obtain final alcohol concentrations of 1, 5, 10, 15, and 20%. The cultures were then incubated at 37 °C for 16 h. The cultivated suspension was serially diluted, and the plate count method was used to determine the bacterial count at 37 °C for 48 h.

### Probiotic Reduction of Biochemical Markers for Alcoholic Liver Injury

For the control group, 1 mL of medium was added, while the alcohol group received 900 μL of medium. In the experimental group, 800 μL per well of silymarin (SML) was added. Except for the control group, 100 μL of alcohol was added to each well, resulting in a final alcohol concentration of 100 mM. Subsequently, 100 μL of SML (30 μg/mL) and LAB suspension were supplemented with the respective groups and cultured for 24 h. The supernatant and cells were then collected for further analysis. The LAB strains used in this experiment were carefully selected for their ability to reduce IL-8 levels and their capacity to withstand a 20% alcohol concentration.

ALT and AST levels were determined by an Aspartate Aminotransferase Activity Colorimetric and an Alanine Aminotransferase Colorimetric Assay Kits (BioVision, Milpitas, California, USA). Cells obtained from the previous experiment were collected and washed twice with ice-cold PBS. Subsequently, 200 μL of ice-cold ALT/AST assay buffer was added to the cells. Homogenization was carried out using a homogenizer, centrifugation (13,000 ×*g*, 10 min) at 4 °C, and collected for analysis.

### LAB Resistance to Simulated Gastrointestinal Conditions and Adhesion Assay

These experimental methods were performed based on Liu et al. [23]. Acid tolerance was at pH 2.0, 3.0 and 7.0 for 3 h. Bile tolerance was determined in MRS broth with and without bile salt. The C2BBel (human colon adenocarcinoma cell; BCRC number: 60182) cell line was purchased from the BCRC. Cells were grown in Dulbecco’s Modified Eagle Medium (DMEM) (Gibco, CA, USA), 10% FBS (Gibco, CA, USA) in a humidified atmosphere of 95% air and 5% CO_2_ at 37 °C. The quantity of LAB cells adhering to the cultured cells was determined using the methodology outlined by Gopal et al. [24].

### Animal Experiments

The animal experiment protocol was approved under the number HK10805 (dated 2019/07/05) by HungKuang University (Taichung, Taiwan). A total of sixty male C57BL/6N mice, aged seven weeks, were obtained from BioLASCO Taiwan Co., Ltd. (Taipei, Taiwan). The mice were divided into groups of ten and housed individually in ventilated cages under controlled conditions: temperature (22 ± 2 °C), relative humidity (55 ± 5%), and a 12-h light/dark cycle. They had ad libitum access to water and a diet that was replenished daily. Mouse weights were recorded weekly to monitor overall growth.

The Lieber-DeCarli liquid alcohol diet, obtained from Medgene CO., LTD. (New Taipei, Taiwan), was used to induce liver injury. It provided 35% of calories from fat, 11% from carbohydrates, 18% from protein, and 36% from either ethanol or isocaloric maltose dextrin (control). Before the experiment began, a one-week acclimation period was implemented, during which the alcohol content was gradually introduced into the diets of both the alcohol and LAB groups to aid in adaptation to the alcohol feed. Once the experiment commenced, the animals were provided with the full amount of alcohol gradually, with maltodextrin added to supplement the caloric intake. The liquid feed had a total caloric content of 1000 kcal/L, with 36% (360 kcal/L) of the calories derived from the complete amount of alcohol (67.3 mL).

The control group received a standard liquid diet using a free-feeding method, while the alcohol group and all LAB groups received the liquid alcohol diet voluntarily. The experiment continued for a duration of 8 weeks. The LAB strains were orally administered daily to the mice at a dosage of 9 log CFU/mL using an oral feeding needle. Retro-orbital blood collection was performed at Weeks 0, 2, 4, 6, and 8 to analyze serum levels of AST, ALT, TG, and total cholesterol. At the conclusion of the eighth week, all mice were weighed and humanely euthanized. Liver specimens were obtained through laparotomy, and approximately 1 cm^3^ tissue pieces were excised from the right lobe. The tissue samples were preserved in 10% neutral formalin, embedded in paraffin, and stained with hematoxylin and eosin (H&E) for histopathological evaluation. GSH content using QuantiChrom™ Glutathione Assay Kit (BioAssay Systems, USA), GPx activity using EnzyChrom™ Glutathione Peroxidase Assay Kit (BioAssay Systems, USA), liver TG levels, and concentrations of cytokines including IL-6, INF-*γ*, and TNF-*α* were assessed. Serum separation was achieved by centrifuging BD SST gel and clot-promoting test tubes at 3500 ×*g* for 15 min at 12 °C using a refrigerated centrifuge. Analysis of AST, ALT, TG, and total cholesterol levels was performed at Dahua Laboratory (Changhua City, Taiwan).

### Enzyme-Linked Immunoassay of IL-6, INF-*γ *and TNF-*α*, Content in Cell Supernatant

Liver tissue weighing 50 mg was combined with 450 μL of cold PBS buffer solution. The resulting mixture was homogenized and subsequently centrifuged (14000 rpm, 10 min, 4 °C). The supernatant was collected for further analysis. ELISA was employed to quantify cytokine levels using BD OptEIATM kits (BD Biosciences, CA, USA) designed for mice cytokines, following the manufacturer’s instructions.

### Statistical Analysis

Statistical analyses were performed using SPSS 23.00 software (IBM Corporation, Armonk, NY, USA). All experiments were conducted in triplicate, with results expressed as mean ± standard deviation (SD). Data were evaluated via one-way analysis of variance (ANOVA), and inter-group differences between means were further analyzed using Duncan’s Multiple Range Test. Statistical significance was assessed through a two-tailed test, with a threshold of *P* < 0.05.

## Results

### Anti-Inflammatory Effects of Probiotics

Following LPS stimulation, IL-6, TNF-*α*, and interferon (IFN)-*γ* production is not observed in HepG2 cells, as IL-6 and TNF-*α* are primarily released by macrophages, while IFN-*γ* is mainly generated by specific subpopulations of activated natural killer cells and helper T1 lymphocytes. Therefore, RAW 264.7 cells were utilized to assess the reduction in IL-6 and TNF-*α* concentrations induced by LAB, while IL-8 production was examined in HepG2 cells. After LPS stimulation of RAW 264.7 cells to induce IL-6 and TNF-*α* production, different LAB strains were separately added and incubated for 24 h, followed by collection of the cell supernatants. ELISA analysis demonstrated that all strains, except (106) 1–1, significantly inhibited IL-6 and TNF-*α* secretion. Subsequently, HepG2 cells were stimulated with LPS to induce IL-8 production, and different LAB strains were added. The results indicated that strain (106) 3–11, strain (106) 1–17, strain (106) 3–14, strain (106) 7–2, strain (107) tau 6–2, strain (107) jia 6–7, strain (107) 6–10, and strain (107) tau 2–4 effectively suppressed IL-8 production (Table 1).

**Table 1:** Anti-inflammatory results of LAB strains based on in vitro cytokine levels

Strain No	TNF-*α* (pg/mL)	IL-6 (pg/mL)	IL-8 (pg/mL)	Strain No	TNF-*α* (pg/mL)	IL-6 (pg/mL)	IL-8 (pg/mL)
Control	273.69^c^	3.18^q^	118.22^c^	(107) tau3-5	1.89^e^	4.75^e^	27.75^n^
LPS (100ng)	469.03^a^	818.16^a^	153.39^b^	(107) tau2-1	1.64^fg^	4.04^lmn^	13.83^t^
(106) 6–1	1.61^fgh^	4.49^ghi^	37.12^j^	(107) 6–10	1.40^ijklm^	4.19^kl^	8.81^v^
(106) 11–6	1.61^fgh^	4.59^fg^	15.09^s^	(107) tau2-8	1.26^mnop^	4.04^lmn^	17.56^r^
(106) 3–9	1.46^ghijkl^	4.44^hij^	32.14^l^	(107) tau6-7	1.46^ghijkl^	4.09^lm^	24.04^o^
(106) 6–4	1.52^fghi^	4.14^lm^	45.14^f^	(106) 6–9	1.28^lmno^	4.04^lmn^	38.54^i^
(106) 8–5	1.31^klmn^	3.99^mn^	37.04^j^	(106) 6–18	1.34^jklm^	3.74^p^	35.33^k^
(106) 3–21②	1.49^ghijk^	3.89^no^	43.45^g^	(106) 1–5	1.64^fg^	4.70^ef^	24.04^o^
(106) 3–11	2.08^d^	5.38^d^	1.11^wx^	(106) 1–4	1.61^fgh^	4.64^ef^	34.22^k^
(106) 1–17	1.61^fgh^	4.70^ef^	1.38^w^	(106) 3–21①	0.93^rs^	2.98^r^	51.86^e^
(106) 3–14	1.73^ef^	4.04^lmn^	1.09^wx^	^†^(106) 1–1	399.15^b^	203.06^b^	177.40^a^
(106) 7–2	1.55^fghi^	4.39^ij^	1.27^wx^	(107) jia6-6	0.81^s^	2.74^s^	38.3^i^
(107) tau6-2	1.49^ghijk^	4.34^ij^	0.88^x^	(107) jia5-7	1.02^qr^	3.13^q^	32.12^l^
(107) jia6-7	1.37^ijklm^	4.39^ij^	0.95^x^	(107) jia6-1	0.79^s^	2.79^s^	32.20^l^
(107) 8–16	1.43^hijklm^	4.34^ij^	12.51^u^	(107) tau1-1	1.08^pqr^	2.69^s^	39.65^h^
(107) jia6-5	1.31^klmn^	4.29^jk^	29.17^m^	(107) tau4-2	1.10^opqr^	3.23^q^	56.54^d^
(107) tau1-3	2.18^d^	5.98^c^	21.89^p^	(107) tau3-7	1.39^ijklm^	4.58^fgh^	19.77^l^
				(107) tau2-4	1.14^nopq^	3.84^op^	8.39^v^

^a,b,c,b,f,g,h,i,j,k,l,m,n,o,p,q,r,s,t,u,v,w,x^Values in the same column with different superscripts mean significant difference (*P* < 0.05)

^†^Strain (106) 1–1 exhibited greater deviations compared to other strains, possibly due to its variable cytokine response to inflammatory stimuli

### Alcohol Tolerance Test

In the presence of alcohol concentrations ranging from 1 to 5%, the bacterial count remained unaffected. However, upon increasing the alcohol concentration to 10%, the following strains exhibited a maintained bacterial count of approximately 8 log CFU/mL: strain (106) 11–6, strain (107) tau 6–2, strain (107) jia 6–7, strain (107) 8–16, strain (107) tau 1–3, strain (107) tau 2–1, strain (107) 6–10, strain (107) tau 2–8, strain (107) jia 5–7, strain (107) tau 1–1, and strain (107) tau 3–7. Subsequently, we selected 27 strains with bacterial counts exceeding 7.5 log CFU/mL at 10% alcohol concentration and subjected them to 15% alcohol. At 15% alcohol, only (107) tau 2–8 demonstrated a bacterial count exceeding 8 log CFU/mL. Among the remaining 26 strains, five exhibited counts below 7.5 log CFU/mL, while 21 maintained counts above 7.5 log CFU/mL. Moving forward, the 22 strains with counts exceeding 7.5 log CFU/mL were exposed to 20% alcohol, revealing that strain (107) tau 2–8 and strain (107) tau 1–1 sustained counts above 6 log CFU/mL, whereas strain (106) 11–6, strain (107) tau 6–2, and strain (107) jia 5–7 maintained counts above 5.5 log CFU/mL. Conversely, the remaining 17 strains exhibited counts below 4 log CFU/mL (Table 2).

**Table 2:** Alcohol tolerance of 33 LAB strains following 16 h of incubation at 37 °C in MRS broth with varying concentrations of ethanol

Strain No	No	1%	5%	10%	15%	20%
	Log CFU/mL					
(106) 6–1	9.39^ef^	9.27^de^	9.14^ef^	7.59^mn^	7.25^h^	–^A^
(106) 11–6	9.36^f^	9.37^c^	9.17^e^	8.24^b^	7.93^b^	5.66^d^
(106) 3–9	9.22^h^	9.10^hi^	8.63^m^	7.60^lmn^	7.51^ef^	5.13^h^
(106) 6–4	9.35^fg^	9.20^efg^	9.15^ef^	7.72^ijk^	7.47^fg^	–
(106) 8–5	9.23^h^	9.25^ef^	8.96^hi^	7.76^hij^	7.53^ef^	–
(106) 3–21-2	9.20^h^	9.19^fgh^	9.30^c^	7.71^ijk^	7.50^ef^	–
(106) 3–11	9.00^k^	9.12^h^	8.77^jkl^	7.36^q^	–	–
(106) 1–17	9.03^k^	9.04^ij^	8.79^jk^	7.48^op^	–	–
(106) 3–14	9.35^fg^	9.18^gh^	9.08^fg^	7.75^hij^	7.38^gh^	–
(106) 7–2	9.07^ijk^	9.12^h^	8.80^j^	7.40^pq^	–	–
(107) tau 6–2	9.56^c^	9.36^c^	8.96^hi^	8.07^cd^	7.81^bc^	5.57^e^
(107) jia 6–7	9.11^i^	8.97^kl^	8.66	8.01^cde^	7.55^ef^	5.39^fg^
(107) 8–16	9.53^c^	9.35^c^	9.22^de^	8.00^cde^	7.47^fg^	–
(107) jia 6–5	9.10^ij^	8.97^kl^	8.79^jk^	7.93^ef^	7.68^de^	5.43^f^
(107) tau 1–3	9.41^def^	9.21^efg^	9.04^gh^	8.00^cde^	7.87^bc^	–
(107) tau 3–5	9.22^h^	9.10^hi^	8.70^klm^	7.79^hi^	7.87^bc^	–
(107) tau 2–1	9.70^a^	9.61^a^	9.58^a^	8.22^b^	7.69^ef^	5.04^i^
(107) 6–10	9.24^h^	9.38^c^	8.99^hi^	8.42^op^	7.77^cd^	6.26^b^
(107) tau 2–8	9.64^b^	9.66^a^	9.45^b^	8.19^b^	8.16^a^	5.32^g^
(107) tau 6–7	9.29^gh^	9.17^gh^	8.90^i^	7.68^jkl^	7.52^ef^	–
(106) 6–9	8.79^l^	8.98^jkl^	8.67^m^	7.53^no^	7.53^ef^	–
(106) 6–18	9.29^gh^	9.12^h^	9.04^gh^	7.81^gh^	7.70^cd^	–
(106) 1–5	9.10^ij^	8.98^jkl^	8.83^j^	7.57^n^	7.54^ef^	–
(106) 1–4	9.03^k^	9.04^ij^	8.82^j^	7.65^klm^	7.29^h^	–
(106) 3–21-1	9.00^k^	8.94^kl^	8.71^klm^	7.37^q^	–	–
(106) 1–1	9.42^de^	9.32^cd^	8.95^i^	7.42^pq^	–	–
(107) jia 6–6	9.04^jk^	8.92^l^	8.69^lm^	7.89^fg^	7.53^ef^	–
(107) jia 5–7	9.13^i^	9.00^jk^	8.82^j^	8.09^c^	7.57^ef^	5.81^c^
(107) jia 6–1	9.09^ijk^	8.94^kl^	8.69^lm^	7.99^de^	7.61^de^	–
(107) tau 1–1	9.45^d^	9.48^b^	9.27^cd^	8.19^b^	7.86^bc^	6.67^a^
(107) tau 4–2	9.29^gh^	8.94^kl^	8.80^j^	7.37^q^	–	–
(107) tau 3–7	9.57^c^	9.32^cd^	9.14^ef^	8.21^b^	7.85^bc^	–
(107) tau 2–4	9.01^k^	9.21^efg^	8.79^jk^	7.81^gh^	7.78^cd^	–

^A^not detected. ^a,b,c,b,f,g,h,i,j,k,l,m,n,o,p,q^Values in the same column with different superscripts mean significant difference (*P* < 0.05)

### Reduction of Alcoholic Liver Injury Biochemical Markers by LAB Strains

**Fig. 1:**
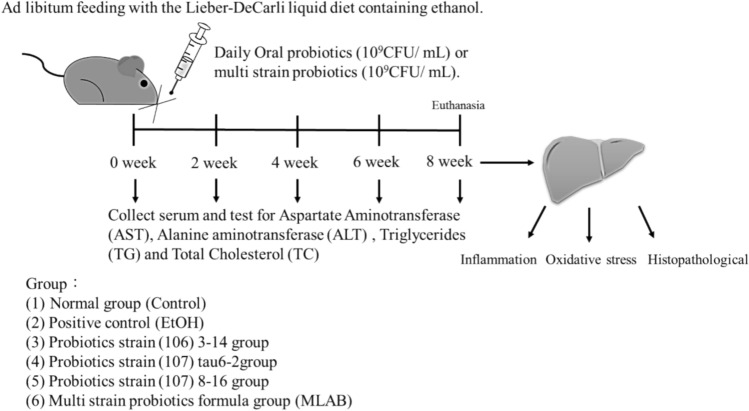
The Flowchart of animal experiments

**Fig. 2:**
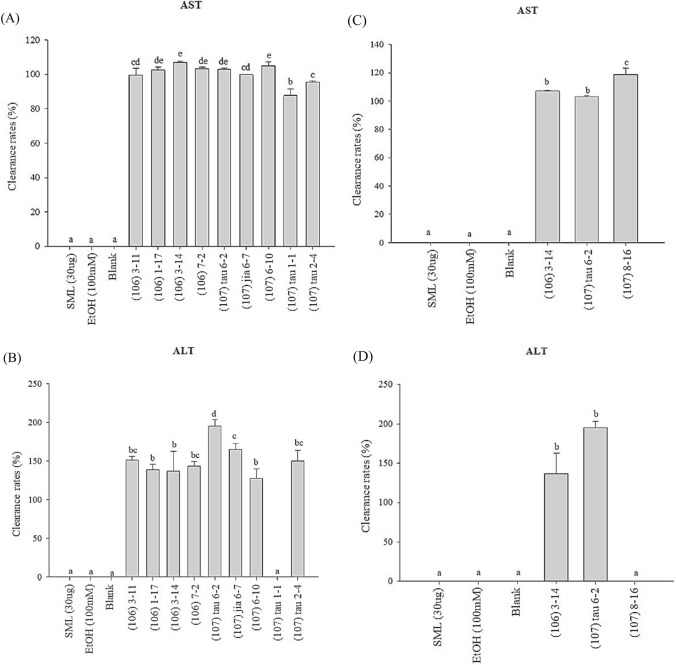
The effect of lactic acid bacteria on the AST scavenging activity of HepG2 cells stimulated by alcohol to produce AST. Different lowercase letters (i.e., a–e) over bars (representing percentages) indicate significant differences in means (*P* < 0.05) using Duncan’s multiple range test. The error bars represent standard deviation

The strains selected for this experiment were specifically chosen for their favourable effects in reducing IL-8 levels and their ability to tolerate 20% alcohol. Notably, the following strains demonstrated significant reductions in AST levels produced by alcohol-stimulated Hep G2 cells: strain (106) 3–11, strain (106) 1–17, strain (106) 3–14, strain (106) 7–2, strain (107) tau 6–2, strain (107) jia 6–7, and strain (107) 6–10 (Fig. 1). The effects of strain (106) 3–14 and strain (107) 6–10 were particularly significant (*P* < 0.05), with clearance rates of 107% and 105%, respectively. Similarly, the following strains effectively decreased the ALT levels produced by alcohol-stimulated Hep G2 cells: strain (106) 3–11, strain (106) 1–17, strain (106) 3–14, strain (106) 7–2, strain (107) tau 6–2, strain (107) jia 6–7, strain (107) 6–10, and strain (107) tau 2–4 (Fig. 2A, B). Notably, the effect of strain (107) tau 6–2 was significant (*P* < 0.05), demonstrating a clearance rate of 195%. Based on these experimental results and strain identification, strain (106) 3–14 and strain (107) tau 6–2 were selected for further animal experiments, with strain (107) 8–16 being the third strain chosen after a subsequent supplementary test. Figure 2C, D displays the clearance rates of AST and ALT produced by alcohol-stimulated Hep G2 cells for these three selected strains.

### Acid and Bile Resistance, and Intestinal Adhesion Ability

As shown in the Table 3, the three strains of *Lactobacillus* demonstrated notable acid tolerance during a 3-h incubation in simulated gastric fluid with a pH of 3.0, as their population remained at or above 8 log CFU/mL. Notably, strain (107) tau 6–2 and strain (107) 8–16 maintained their initial bacterial counts, indicating their resistance to gastric acidity. Subsequently, the *Lactobacillus* strains, after the 3-h exposure to pH 3.0 simulated gastric fluids, were subjected to a phosphate solution containing 0.15% bile salts. Among these strains, (107) 8–16 exhibited the highest resistance to bile salts, with a bacterial count of 9.28 log CFU/ mL. It was closely followed by strain (106) 3–14, with a bacterial count of 8.49 ± 0.02 log CFU/ mL. The strain (106) 3–14, strain (107) tau 6–2, and strain (107) 8–16 exhibited an average adherence capacity of 29 ± 16.08, 34.4 ± 16.33, and 36.8 ± 15.37 bacterial cells per C2BBel cell, respectively. According to previous studies, it has been reported that each cell can adhere to more than 15 bacterial cells, indicating their adhesive capacity [25].

**Table 3:** Results of LAB strain resistance to simulated gastrointestinal conditions and adhesion assay

Probiotic characteristics		LAB strains		
		(106) 3–14	(107) tau6-2	(107) 8–16
Acid tolerance (log CFU/ mL)	0 h	9.54 ± 0.02^a^	8.37 ± 0.23^b^	9.22 ± 0.02^a^
	1.5 h	9.22 ± 0.02^b^	8.46 ± 0.06^c^	9.55 ± 0.03^a^
	3 h	8.89 ± 0.16^b^	8.2 ± 0.04^c^	9.41 ± 0.02^a^
Bile salt tolerance (log CFU/mL)	0 h	8.55 ± 0.14^b^	8.13 ± 0.02^c^	9.32 ± 0.03^a^
	1.5 h	8.61 ± 0.05^b^	7.78 ± 0.02^c^	9.4 ± 0.02^a^
	3 h	8.49 ± 0.02^b^	7.49 ± 0.18^c^	9.28 ± 0^a^
Adherence of LAB to C2BBel cells (CFU/ cell)		29 ± 16.08^a^	34.4 ± 16.33^a^	36.8 ± 15.37^a^

^a,b,c^Values in the same column with different superscripts mean significant difference (*P* < 0.05)

### Animal Experiments

At the strain selection, the primary criterion was to inhibit the IL-8 inflammatory response. Strains capable of reducing levels to below 10 pg/ mL were selected for further analysis of AST and ALT. In experiments conducted on HepG2 cells stimulated by alcohol, the most effective strains in reducing AST and ALT levels were strain (106) 3–14 and strain (107) tau 6–2, with clearance rates of 107% and 195%, respectively. Subsequent supplementation tests, including acid and bile resistance as well as adhesion assays, identified strain (107) 8–16 as the most effective strain.

**Fig. 3:**
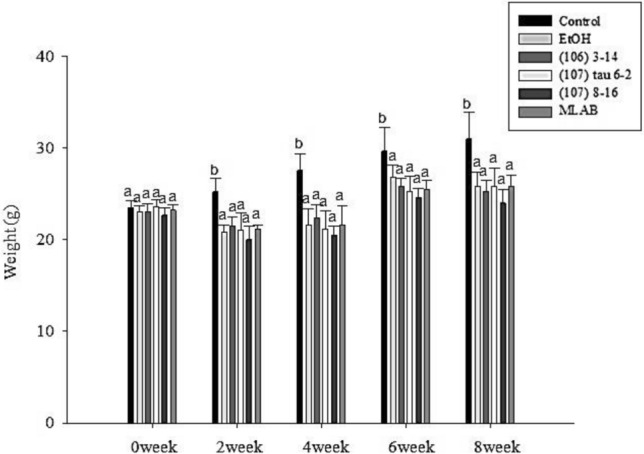
Changes in body weight of C57BL/6N mice in each group during the eight-week experiment (n = 10). Different lowercase letters (i.e., a or b) over bars (representing weight) indicate significant differences in means (*P* < 0.05) using Duncan’s multiple range test. The error bars represent standard deviation

Figure 3 illustrates the changes in body weight for mice fed with general liquid feed and liquid alcohol feed over the 8-week feeding period. Throughout the study, the control group showed an increase in body weight, while the alcohol group exhibited decreased body weight, and the LAB groups showed minimal weight change. Notably, despite being on a normal diet, the control group had significantly higher body weight compared to both the alcohol and LAB groups (*P* < 0.05). This disparity can be attributed to weight loss resulting from alcoholic hepatitis.

**Fig. 4:**
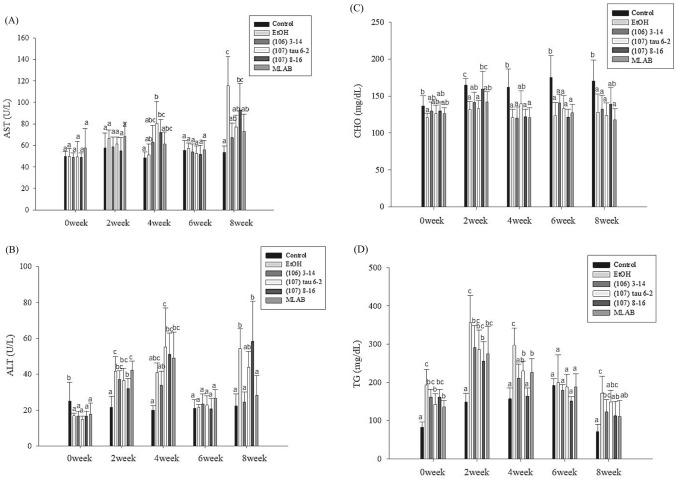
Effects of lactic acid bacteria on **A** AST, **B** ALT activity, **C** cholesterol and **D** triglycerides levels in serum of C57BL/6N mice fed a liquid alcohol diet for eight weeks (n = 10). Different lowercase letters (i.e., a-c) over bars indicate significant differences in means (*P* < 0.05) using Duncan’s multiple range test. The error bars represent standard deviation

Figure 4A displays the changes in serum AST levels among the different groups of C57BL/6N mice during the 8-week experiment. The control group demonstrated negligible fluctuations in serum AST levels throughout the study period. In contrast, the alcohol group exhibited a substantial increase in AST levels at Week 8 (53%), significantly higher than the control group (*P* < 0.05). While the AST levels of mice fed with various LAB strains and a combination of LAB also increased during the study period, they did not significantly differ from the alcohol group before Week 8. However, at Week 8, the serum AST levels of groups fed with a single strain (106) 3–14, strain (107) tau 6–2, and the strain combination were significantly lower than the alcohol group (*P* < 0.05), showing reductions of 42%, 33%, and 37%, respectively. The observed increase in serum AST levels in C57BL/6N mice fed an alcohol diet may be attributed to liver function impairment caused by alcohol consumption, which leads to the release of AST into the bloodstream [26].

Figure 4B depicts the changes in serum ALT levels among the different groups of C57BL/6N mice over the 8-week period. The control group exhibited minimal changes in serum ALT levels during the 8-week period. In contrast, the alcohol group demonstrated a substantial increase in ALT levels as the weeks progressed, significantly differing (*P* < 0.05) from the control group and LAB groups at Week 8. Notably, the single strain (106) 3–14 and the combination of strains exhibited stronger effects, reducing ALT levels by 54% and 48%, respectively. The elevation in serum ALT levels in C57BL/6N mice fed an alcohol diet can be attributed to alcohol-induced liver damage, resulting in the release of ALT into the bloodstream [26]. While other factors may contribute to the increase in AST values, ALT is directly associated with liver function, and higher concentrations of ALT in the blood indicate more severe liver damage. Figure 3 demonstrates that feeding a single strain (106) 3–14 and a combination of strains effectively reduces serum ALT levels in mice subjected to an alcohol diet, thus mitigating liver injury.

### Changes in Total Cholesterol Content and TG Levels in Serum

Figure 4C depicts the changes in serum cholesterol concentration among different groups of C57BL/6N mice fed a liquid alcohol diet during the 8-week experiment. The control group showed an increase in serum cholesterol levels throughout the feeding period, while the single-strain LAB and combined LAB groups exhibited minimal changes in serum total cholesterol over the 8 weeks. However, no significant differences were observed between the groups. These findings indicate that the consumption of single-strain LAB and combined LAB does not alleviate the alcohol-induced increase in total cholesterol levels.

Figure 4D demonstrates the changes in serum TG levels among different groups of C57BL/6N mice during the study period. The control groups showed minimal changes in serum TG levels throughout the 8-week period. In contrast, the groups fed the alcohol diet exhibited an increase in serum TG levels over time. Among the single-strain LAB and combined LAB groups, there was a substantial decrease in TG values at Week 4, with reductions of 28.9, 22.7, 44.8, and 23.9%, respectively. At Week 6, due to the poor alcohol tolerance of the mice in the alcohol group, there was an increased death rate. To ensure data integrity, the alcohol concentration was reduced to improve their survival rate, resulting in a decrease in serum TG values at Week 6. However, at Week 8, reduced serum TG values were observed in mice fed with single-strain LAB and combined LAB. The reductions in the single strain (107) 8–16 and combined strain groups (35.4 and 34.4%, respectively) were significantly different from the alcohol group (*P* < 0.05), indicating that LAB can effectively counteract the alcohol-induced increase in serum TG levels.

### Expression of TNF-*α*, IL-6 and IFN-*γ* in the Liver

**Fig. 5:**
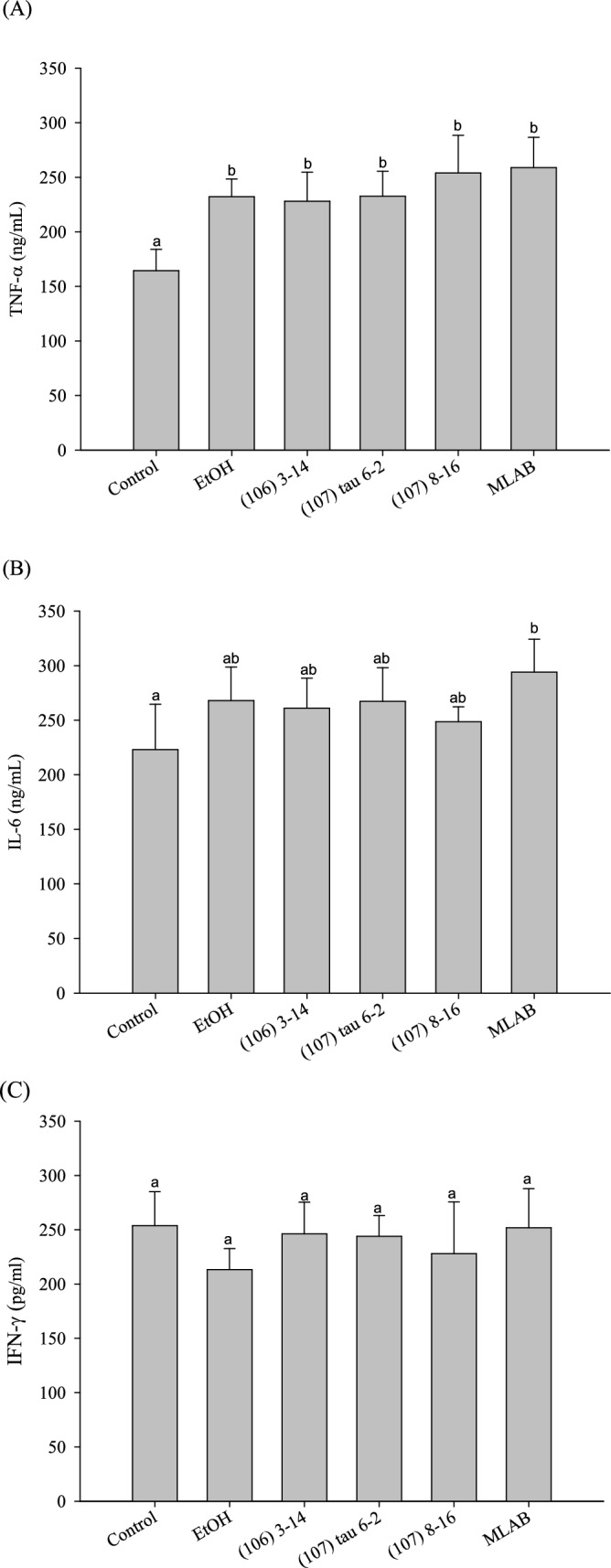
Effects of lactic acid bacteria feeding on **A** TNF-*α*, **B** IL-6 and **C** IFN-*γ* secretion in the liver of C57BL/6N mice fed a liquid alcohol diet for eight weeks (n = 10). Different lowercase letters (i.e., a or b) over bars (representing cytokine levels) indicate significant differences in means (*P* < 0.05) using Duncan’s multiple range test. The error bars represent standard deviation

Figure 5A illustrates the changes in hepatic TNF-*α* expression after 8 weeks of feeding single-strain LAB and combined LAB. The alcohol diet group and every LAB experimental group showed significant increases in liver TNF-*α* content compared to the control group (*P* < 0.05), indicating an elevation in hepatitis markers caused by the alcohol diet. However, there were no distinct differences observed between the alcohol group and the LAB experimental groups, possibly due to the excessive severity of the inflammatory response induced by the alcohol diet, rendering the LAB unable to effectively mitigate inflammation.

Figure 5B presents the changes in hepatic IL-6 expression for the single-strain LAB and combined LAB feeding groups after 8 weeks. The alcohol diet group and LAB experimental groups exhibited increased liver IL-6 content compared to the control group. However, no significant differences were observed between these groups.

Figure 5C demonstrates the changes in hepatic IFN-*γ* expression after 8 weeks of feeding single-strain LAB and combined LAB. Although the liver IFN-*γ* contents of single strain (106) 3–14 and strain (107) tau 6–2, as well as the combined LAB, were higher than those of the alcohol group, the differences between the alcohol diet group, LAB experimental groups, and the control group were not statistically significant.

### Changes in TG and GSH Content in the Liver

**Fig. 6:**
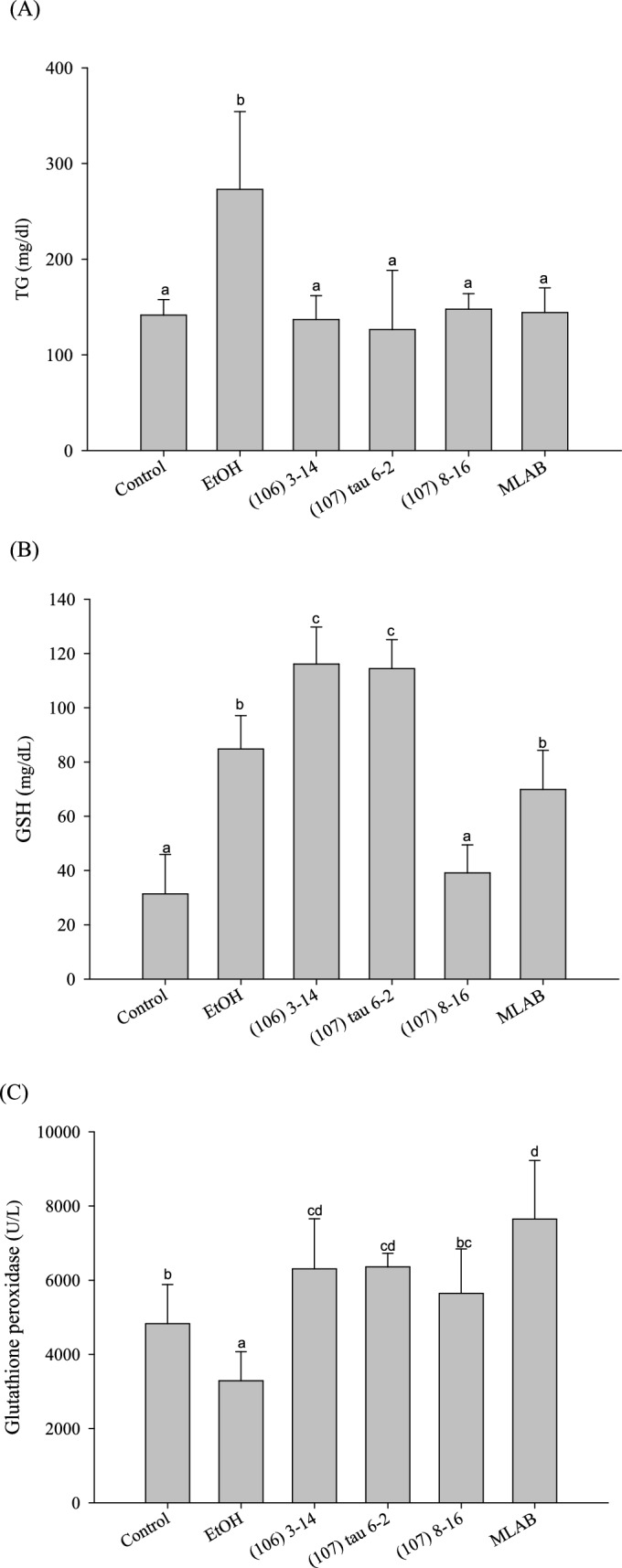
Effects of feeding lactic acid bacteria for eight weeks on **A** triglyceride, **B** glutathione levels and **C** glutathione peroxidase activity in the liver of C57BL/6N mice fed a liquid alcohol diet (n = 10). Different lowercase letters (i.e., a-d) over bars indicate significant differences in means (*P* < 0.05) using Duncan’s multiple range test. The error bars represent standard deviation

Figure 6A depicts the changes in liver TG levels after 8 weeks of single-strain LAB and combined LAB feeding. The alcohol diet group (273.08 ± 81.15 mg/dL) exhibited a substantial increase in liver TG levels by 48.2% after 8 weeks, which significantly differed from the control group (141.55 ± 16.26 mg/dL) (*P* < 0.05). Conversely, feeding with single-strain LAB and combined LAB resulted in significant reductions in liver TG levels, with decreases of 49.8% [(106) 3–14137.03 ± 24.87 mg/dL], 53.7% [strain (107) 3–14, 126.37 ± 61.88 mg/dL], 45.9% [strain (107) 8–16, 147.86 ± 16.16 mg/dL], and 47.2% (MLAB, 144.24 ± 25.67 mg/dL), respectively.

Figure 6B presents the liver GSH content. The alcohol diet group (84.78 ± 12.3 mg/dl) displayed a significant difference in GSH content compared to the control group (31.41 ± 14.47 mg/dL), exhibiting a 63% increase in GSH content. Among the single-strain LAB and combined LAB groups, there were no significant differences between the single strain (107) 8–16 and control groups (*P* < 0.05) or between the combined LAB and alcohol groups (*P* < 0.05). However, there was a significant difference between the combined LAB and control groups (*P* < 0.05), with the combined LAB group (69.89 ± 14.43 mg/dL) showing a 55% increase in GSH content. Furthermore, compared to the control and alcohol groups, the single-strain (106) 3–14 (116.1 ± 13.63 mg/dL) and strain (107) tau 6–2 (114.41 ± 10.68 mg/dL) groups exhibited substantial increases (*P* < 0.05), surpassing the alcohol group (84.78 ± 12.3 mg/dL) by 37 and 35%, respectively.

### Antioxidant Enzyme Activity of Glutathione Peroxidise (GPx)

Figure 6C illustrates the enzymatic activity of GPx in the liver of the combined LAB group after 8 weeks. The GPx activity in the alcohol group (3289.24 ± 785.79 U/L) decreased by 31.9% compared to the control (4828.62 ± 1054 U/L) group. Significant differences (*P* < 0.05) were observed when comparing the single LAB strain (106) 3–14 (6306.37 ± 1349.02 U/L), strain (107) tau 6–2 (6356.48 ± 368.65 U/L), strain (107) 8–16 (5643.42 ± 1199.19 U/L), and the combined LAB groups (7648.83 ± 1580.45 U/L) with the control group and alcohol group. In comparison to the alcohol group, they exhibited increases of 91.7, 93.3, 71.6, and 132.5%, respectively.

#### Tissue Slices

**Fig. 7:**
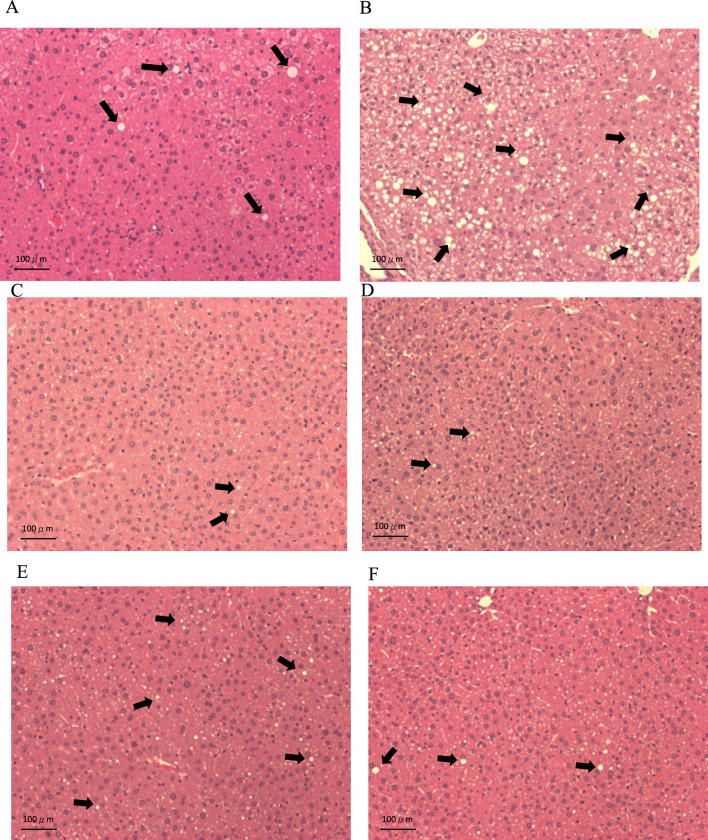
Results of H&E staining of liver tissues from C57BL/6N mice fed alcohol diets for 8 weeks, using a general liquid feed and LAB: **A** liquid general feed control group, **B** liquid alcohol feed, **C** (106) 3–14, **D** (107) tau 6–2, **E** (107) 8–16 and **F** Multi-strain LAB liver tissue injury in C57BL/6N mice after eight weeks. Black arrows represent the accumulation of fat droplets. Scale bar, 100 µm

Figure 7 depicts the results of H&E staining of liver tissues from C57BL/6N mice fed alcohol diets for 8 weeks, using a general liquid feed and LAB. Figure 7A shows the control group fed a general liquid feed, Fig. 7B represents the alcohol group, and Fig. 7C, D, E display the groups fed with single strain (106) 3–14, strain (107) tau 6–2, and strain (107) 8–16, as well as the combined strains, respectively. The control and LAB groups exhibited no noticeable accumulation of fat/oil droplets and displayed intact cell nuclei. Remarkably, the single strain (106) 3–14 exhibited a particularly exceptional effect. Although some fat/oil droplets were present in strain (107) tau 6–2, strain (107) 8–16, and the combined strains, the reduction was substantial difference compared to the alcohol group. The alcohol group displayed conspicuous accumulation of fat/oil droplets, nearly covering the entire section. These findings indicate that LAB feeding mitigates and reduces hepatic fat accumulation, thereby preventing fatty liver. The API kit was used for strain identification, strain (107) tau 6–2 was identified as *L. paracasei*, strain (107) 8–16 was identified as *L. plantarum*, and strain (106) 3–14 was identified as *Pidiococcus pentosaceus*.

## Discussion

In order to elicit beneficial effects, the viability of bacterial strains is a fundamental requirement for probiotics. To exert positive effects in the intestinal tract, probiotics must successfully navigate the acidic environment of the stomach and maintain their viability. The pH value of a healthy human stomach is approximately 2–2.5 [27], typically occurring during prolonged gastric emptying without food or liquid intake. However, the pH value of gastric acid can vary depending on the type and timing of food consumption, typically ranging from pH 1.5 to 4.58. The duration of food retention varies based on food type and quantity, with an average retention time of about 2–3 h [28].

In this acid tolerance test, simulated gastric fluid was prepared by suspending the bacterial strains in a PBS solution containing pepsin (pH 3). For the bile salt tolerance test, the simulated intestinal fluid contained both ox bile and trypsin. Ayyash et al. (2021) reviewed studies evaluating simulated gastric survival and found that most studies only assessed low pH conditions without the addition of pepsin, and the exposure time was relatively short, typically 1 to 2 h [29]. These methods raise concerns about survival rate accuracy, as the pH range in the human stomach varies from 1.5 in the fasting state to 4.5 after meals, and food digestion may take up to 3 h. Additionally, factors such as bile salts, trypsin, and high pH in the intestines create additional antimicrobial stress on ingested probiotics [29]. Therefore, in this study, bacteria were first treated with low pH gastric acid and pepsin, followed by exposure to high pH intestinal fluid containing bile salts and trypsin, to more closely simulate human gastrointestinal conditions. Therefore, in this study, bacteria were first treated with low pH gastric acid and pepsin, followed by exposure to high pH intestinal fluid containing bile salts and trypsin, to more closely simulate human gastrointestinal conditions.

Probiotics require a carrier resembling food to enter the human body. In such cases, food intake elevates the pH value, and the food matrix acts as a protective barrier, shielding bacteria from destruction by stomach acid [30]. Resistance to bile salts is also a crucial aspect when investigating probiotic characteristics, as the small intestine and colon harbour a significant concentration of bile salts, which possess toxicity and pose a threat to bacteria [31]. Bile salts can increase the permeability of bacterial cell membranes, making it essential that probiotic strain selection considers their survival rates in such conditions [32].

Alcoholic liver disease primarily arises from the excessive production of LPS indirectly triggered by alcohol consumption. Consequently, LPS stimulates the activation of liver Kupffer cells and leads to excessive TNF-*α* secretion, exacerbating liver injury [17]. Similar to TNF-*α*, the inflammatory response induced by the alcohol diet was overly severe, resulting in the limited reduction of IL-6 secretion by feeding LAB. Involved in liver tissue homeostasis, liver regeneration, antiviral defense, and metabolic regulation under normal physiological conditions is IL-6. However, detrimental and even leading to liver cancer can be the continuous activation of the IL-6 signalling pathway. Research has shown that IL-6 promotes the liver’s synthesis of acute-phase proteins, which are secreted by neutrophils, monocytes, and macrophages in response to TLR stimulation, such as LPS stimulation. Activated bone marrow cells also release inflammatory cytokines (IL-1 and TNF-*α*), triggering the production and secretion of IL-6 in large quantities by other cells, including endothelial cells and fibroblasts, thus establishing positive feedforward control [33]. Studies have indicated that cytokines, including interferon IFN-*γ*, hepatocyte growth factor, and IL-10, possess anti-fibrotic properties in the liver. IFN-*γ* exhibits antiviral effects, activates phagocytes, and induces the expression of major histocompatibility complex molecules [34]. Therefore, although LAB supplementation increased liver IFN-*γ* content, the damage caused by the alcohol diet in this study exceeded the reparative capacity of LAB, resulting in the absence of significant differences.

Produced as a result of ethanol metabolism are large amounts of ROS, which are the primary contributors to oxidative stress and the accumulation of lipid peroxides in the liver. Therefore, a potent direction for treating alcoholic liver disease (ALD) is regulating the antioxidant level in the liver. Exogenous supplementation with GSH treatment boosted both glutathione levels and the antioxidant capacity in hepatocytes, thereby accelerating liver’s regeneration [35]. GSH is an essential antioxidant responsible for scavenging free radicals. It is present in large quantities in the cytoplasm, nuclei, and mitochondria of organisms. The liver and kidneys of humans have particularly high GSH content, with the liver having the highest levels [36–38]. Therefore, we infer that feeding strain (106) 3–14 and strain (107) tau 6–2 for 8 weeks can enhance hepatic GSH content, thereby bolstering the host’s antioxidant capacity.

Rats exhibiting severe liver pathology accompanied by peak levels of lipid peroxidation show decreases in CuZnSOD, GPX, and CAT [39]. Antioxidant enzymes, particularly glutathione peroxidase (GSH-Px) and superoxide dismutase (SOD), can decrease the levels of free radicals and the formation of reactive oxygen species in cells. They are essential for reducing lipid peroxidation and preserving the integrity and functionality of cell membranes [40]. GPx is a protease composed of four identical subunits, and each subunit has a selenium atom as a reaction center on its surface. GPx, along with GSH and GSSG (Glutathione disulphide), collaborates in reducing peroxidized chemical molecules [41]. Our findings suggest that an 8-week LAB supplementation can enhance GPx activity, which, coupled with the increase in GSH content, further strengthens the antioxidant capacity. The development of fatty liver is strongly associated with lipid peroxidation and oxidative stress [42].

Fatty liver disease, including alcoholic fatty liver and non-alcoholic fatty liver, is a prevalent chronic liver disease worldwide, including in Taiwan. The incidence rate of fatty liver in Taiwan is high, reaching 40% according to a survey by the Taiwan Liver Disease Prevention Academic Foundation. Chronic liver disease can be caused by excessive alcohol consumption, with liver diseases and related deaths attributed to alcohol abuse increasing globally each year [43]. Recent studies have focused on probiotics such as *L. acidophilus*, *L. plantarum*, *L. rhamnosus* GG (LGG), *L. reuteri*, *L. fermentum*, *L. salivarius*, *L. johnsonii*, and *Pediococcus pentosaceus* [18, 19, 44, 45]. Alcohol disrupts the gut microbiome, alters the gastrointestinal barrier, and affects various digestive functions such as mucosal resistance [46]. The gut microbiota’s role in alcohol-related conditions is increasingly recognized [46, 47]. Specific bacteria, such as *Lactobacillus rhamnosus* GG, can modulate the gastrointestinal microbiome to modify the course of alcoholic liver disease and other disorders [48].

AST and ALT levels are among the most indicative markers of liver health and hepatotoxicity [49]. AST predominantly resides in the cytoplasm and mitochondria of hepatocytes, while ALT is primarily located in the cytoplasm, leading to increased ALT and AST levels in the body when hepatocytes are damaged [50]. Following lipid peroxidation of liver tissues, the permeability of liver membranes changes, resulting in a sharp increase in ALP levels within liver cells [51]. Human clinical trials have demonstrated that patients receiving probiotic treatment exhibited significantly reduced AST and ALT levels by the end of treatment compared to those who received only standard therapy [52]. Our experiments have shown that feeding single strain (106) 3–14 and combinations of bacteria can effectively improve serum ALT and AST values under alcoholic diet, thereby improving liver damage.

Liver injury can lead to the buildup of unsaturated fats in the liver, subsequently raising triglyceride (TG) levels. Additionally, both TG and total cholesterol (TC) levels serve as indicators of lipid peroxidation in the liver, as this process increases TG and TC levels in the body [53]. Long-term and chronic alcohol consumption are implicated in the onset of fatty liver, primarily due to the accumulation of triglycerides and cholesterol in the liver. Although fatty liver itself does not cause harm to the human body, it serves as a significant warning sign [54]. Alcoholic fatty liver has a particular likelihood of progressing to liver fibrosis and cirrhosis in later stages [55]. Chuang et al. (2016) experiment result shows *Lactobacillus salivarius* significantly inhibited in animal models of acute alcohol exposure serum TG levels by 39.2% [42]. Our current research results indicate that the single strain of lactic acid bacteria and the combined lactic acid bacteria group selected for animal testing can significantly reduce the triglyceride content in both the liver and serum.

In this experimental study, LAB strains isolated from winemaking byproducts have good acid and bile resistance, and intestinal adhesion ability. LAB supplementation significantly reduced hepatic fat accumulation and improved liver function indices, indicating a reduction in liver damage. Additionally, LAB supplementation resulted in increased GSH content and enhanced antioxidant enzyme activity, particularly GPx, thereby strengthening the host’s antioxidant capacity and mitigating free radical-induced damage. Primarily functions of strain (106) 3–14 in antioxidation and anti-inflammation. It reduces serum AST/ALT levels, increases hepatic GSH content and glutathione peroxidase (GPx) activity, decreases inflammatory markers TNF-*α* and IL-6, and reduces hepatic fat accumulation. Mainly effects of strain (107) tau 6–2 in hepatic lipid metabolism and enhances antioxidative capacity. While it does not lower serum ALT levels, it reduces AST levels, increases hepatic GSH content and GPx activity, and decreases hepatic triglyceride (TG) accumulation. Primarily impacts of strain (107) 8–16 in serum lipid metabolism. It increases GPx activity, lowers serum triglyceride levels, and reduces hepatic fat accumulation. Three-strains combination are demonstrated synergistic effects. In addition to lowering serum AST/ALT levels, it exhibits the strongest antioxidative activity (GPx increased by 58.4%), reduces hepatic triglyceride content, and enhances tissue repair capacity. In conclusion, LAB supplementation effectively attenuated alcoholic fatty liver and liver injuries in this study.

## Data Availability

All data generated or analyzed during this study are included in this published article.

## References

[1] Patel S, Mandaliya D, Seshadri S (2022) Colonic microflora protagonist of liver metabolism and gut permeability: study on mice model. Indian J Microbiol 62:540–549. 10.1007/s12088-022-01032-x 36458218 10.1007/s12088-022-01032-x PMC9705630

[2] Sookoian S, Pirola CJ (2015) Liver enzymes, metabolomics and genome-wide association studies: from systems biology to the personalized medicine. World J Gastroenterol 21:711–725. 10.3748/wjg.v21.i3.711 25624707 10.3748/wjg.v21.i3.711 PMC4299326

[3] Giannini EG, Testa R, Savarino V (2005) Liver enzyme alteration: a guide for clinicians. CMAJ 172:367–379. 10.1503/cmaj.1040752 15684121 10.1503/cmaj.1040752 PMC545762

[4] Fukui H, Brauner B, Bode JC, Bode C (1991) Plasma endotoxin concentrations in patients with alcoholic and non-alcoholic liver disease: reevaluation with an improved chromogenic assay. J Hepatol 12:162–169. 10.1016/0168-8278(91)90933-3 2050995 10.1016/0168-8278(91)90933-3

[5] Bishehsari F, Magno E, Swanson G, Desai V, Voigt RM, Forsyth CB, Keshavarzian A (2017) Alcohol and gut-derived inflammation. Alcohol Res 38:163–171 28988571 10.35946/arcr.v38.2.02 PMC5513683

[6] Purohit V, Bode JC, Bode C, Brenner DA, Choudhry MA, Hamilton F, Kang YJ, Keshavarzian A, Rao R, Sartor RB, Swanson C, Turner JR (2008) Alcohol, intestinal bacterial growth, intestinal permeability to endotoxin, and medical consequences: summary of a symposium. Alcohol 42:349–361. 10.1016/j.alcohol.2008.03.131 18504085 10.1016/j.alcohol.2008.03.131 PMC2614138

[7] Rao RK, Seth A, Sheth P (2004) Recent advances in alcoholic liver disease I. Role of intestinal permeability and endotoxemia in alcoholic liver disease. Am J Physiol Gastrointest Liver Physiol 286:G881–G884. 10.1152/ajpgi.00006.2004 15132946 10.1152/ajpgi.00006.2004

[8] Dou L, Shi X, He X, Gao Y (2020) Macrophage phenotype and function in liver disorder. Front Immunol 10:3112. 10.3389/fimmu.2019.03112 32047496 10.3389/fimmu.2019.03112 PMC6997484

[9] Slevin E, Baiocchi L, Wu N, Ekser B, Sato K, Lin E, Ceci L, Chen L, Lorenzo SR, Xu W, Kyritsi K, Meadows V, Zhou T, Kundu D, Han Y, Kennedy L, Glaser S, Francis H, Alpini G, Meng F (2020) Kupffer cells: inflammation pathways and cell-cell interactions in alcohol-associated liver disease. Am J Pathol 190:2185–2193. 10.1016/j.ajpath.2020.08.014 32919978 10.1016/j.ajpath.2020.08.014 PMC7587925

[10] Bharti A, Sharma I, Mahajan R, Langer S, Kapoor N (2024) From cirrhosis to the dysbiosis (A loop of cure or complications?). Indian J Microbiol 64:810–820. 10.1007/s12088-024-01267-w 39282182 10.1007/s12088-024-01267-w PMC11399373

[11] Chayanupatkul M, Somanawat K, Chuaypen N, Klaikeaw N, Wanpiyarat N, Siriviriyakul P, Tumwasorn S, Werawatganon D (2022) Probiotics and their beneficial effects on alcohol-induced liver injury in a rat model: the role of fecal microbiota. BMC Compl Med Ther 22:168. 10.1186/s12906-022-03643-9 10.1186/s12906-022-03643-9 PMC9215017 35733194

[12] Kurose I, Miura S, Higuchi H, Watanabe N, Kamegaya Y, Takaishi M, Tomita K, Fukumura D, Kato S, Ishii H (1996) Increased nitric oxide synthase activity as a cause of mitochondrial dysfunction in rat hepatocytes: roles for tumor necrosis factor alpha. Hepatology 24:1185–1192. 10.1002/hep.510240534 8903396 10.1002/hep.510240534

[13] Lobo V, Patil A, Phatak A, Chandra N (2010) Free radicals, antioxidants and functional foods: impact on human health. Pharmacogn Rev 4:118–126. 10.4103/0973-7847.70902 22228951 10.4103/0973-7847.70902 PMC3249911

[14] Pizzino G, Irrera N, Cucinotta M, Pallio G, Mannino F, Arcoraci V, Squadrito F, Altavilla D, Bitto A (2017) Oxidative stress: harms and benefits for human health. Oxid Med Cell Longev 2017:8416763. 10.1155/2017/8416763 28819546 10.1155/2017/8416763 PMC5551541

[15] Li F, Duan K, Wang C, McClain C, Feng W (2016) Probiotics and alcoholic liver disease: treatment and potential mechanisms. Gastroenterol Res Pract 2016:5491465. 10.1155/2016/5491465 26839540 10.1155/2016/5491465 PMC4709639

[16] Fuenzalida C, Dufeu MS, Poniachik J, Roblero JP, Valenzuela-Pérez L, Beltrán CJ (2021) Probiotics-based treatment as an integral approach for alcohol use disorder in alcoholic liver disease. Front Pharmacol 12:729950. 10.3389/fphar.2021.729950 34630107 10.3389/fphar.2021.729950 PMC8497569

[17] Segawa S, Wakita Y, Hirata H, Watari J (2008) Oral administration of heat-killed *Lactobacillus brevis* SBC8803 ameliorates alcoholic liver disease in ethanol-containing diet-fed C57BL/6N mice. Int J Food Microbiol 128:371–377. 10.1016/j.ijfoodmicro.2008.09.023 18976829 10.1016/j.ijfoodmicro.2008.09.023

[18] Gan Y, Tong J, Zhou X, Long X, Pan Y, Liu W, Zhao X (2021) Hepatoprotective effect of *Lactobacillus plantarum* HFY09 on ethanol-induced liver injury in mice. Front Nutr 8:684588. 10.3389/fnut.2021.684588 34249992 10.3389/fnut.2021.684588 PMC8264191

[19] Hsieh PS, Chen CW, Kuo YW, Ho HH (2021) *Lactobacillus* spp. reduces ethanol-induced liver oxidative stress and inflammation in a mouse model of alcoholic steatohepatitis. Exp Ther Med 21:188. 10.3892/etm.2021.9619 33488797 10.3892/etm.2021.9619 PMC7812587

[20] Kurutas EB (2016) The importance of antioxidants which play the role in cellular response against oxidative/nitrosative stress: current state. Nutr J 15:71. 10.1186/s12937-016-0186-5 27456681 10.1186/s12937-016-0186-5 PMC4960740

[21] Kanmani P, Kim H (2018) Protective effects of lactic acid bacteria against tlr4 induced inflammatory response in hepatoma HepG2 cells through modulation of toll-like receptor negative regulators of mitogen-activated protein kinase and NF-κB signaling. Front Immunol 9:1537. 10.3389/fimmu.2018.01537 30022981 10.3389/fimmu.2018.01537 PMC6039550

[22] Hsieh MW, Chen HY, Tsai CC (2021) Screening and evaluation of purine-nucleoside-degrading lactic acid bacteria isolated from winemaking byproducts in vitro and their uric acid-lowering effects in vivo. Fermentation 7:74. 10.3390/fermentation7020074

[23] Liu YH, Ho CH, Huang CC, Tsai CC (2016) Inhibitory effect of lactic acid bacteria on uropathogenic *Escherichia coli*-induced urinary tract infections. J Prob Health 4:144–150. 10.4172/2329-8901.1000144

[24] Gopal PK, Prasad J, Smart J, Gill HS (2001) *In vitro* adherence properties of *Lactobacillus rhamnosus* DR20 and *Bifidobacterium lactis* DR10 strains and their antagonistic activity against an enterotoxigenic *Escherichia coli*. Int J Food Microbiol 67:207–216. 10.1016/s0168-1605(01)00440-8 11518430 10.1016/s0168-1605(01)00440-8

[25] Pedersen K, Tannock GW (1989) Colonization of the porcine gastrointestinal tract by lactobacilli. Appl Environ Microbiol 55:279–283. 10.1128/aem.55.2.279-283.1989 2719474 10.1128/aem.55.2.279-283.1989 PMC184101

[26] Kirpich IA, Solovieva NV, Leikhter SN, Shidakova NA, Lebedeva OV, Sidorov PI (2008) Probiotics restore bowel flora and improve liver enzymes in human alcohol-induced liver injury: a pilot study. Alcohol 42:675–682. 10.1016/j.alcohol.2008.08.006 19038698 10.1016/j.alcohol.2008.08.006 PMC2630703

[27] Fernandez MF, Boris S, Barbe’s C (2003) Probiotic properties of human *Lactobacilli* strains to be used in the gastrointestinal tract. J Appl Microbiol 94:449–455 12588553 10.1046/j.1365-2672.2003.01850.x

[28] Insel P, Turner RE, Ross D (2001) Nutrition. digestion and absorption. Jones and Bartlett Publishers, Massachusetts, USA, pp 64–97

[29] Ayyash MM, Abdalla AK, AlKalbani NS, Baig MA, Turner MS, Liu SQ, Shah NP (2021) Invited review: characterization of new probiotics from dairy and nondairy products-insights into acid tolerance, bile metabolism and tolerance, and adhesion capability. J Dairy Sci 104:8363–8379. 10.3168/jds.2021-20398 33934857 10.3168/jds.2021-20398

[30] Hojjati M, Behabahani BA, Falah F (2020) Aggregation, adherence, anti-adhesion and antagonistic activity properties relating to surface charge of probiotic *Lactobacillus brevis* gp104 against *Staphylococcus aureus*. Microb Pathog 147:104420. 10.1016/j.micpath.2020.104420 32763413 10.1016/j.micpath.2020.104420

[31] Tokatli M, Gulgor G, Bagder Elmaci S, Arslankoz Isleyen N, Ozcelik F (2015) In vitro properties of potential probiotic indigenous lactic acid bacteria originating from traditional pickles. Biomed Res Inter 2015:315819. 10.1155/2015/315819 10.1155/2015/315819 PMC4460932 26101771

[32] Aarti C, Khusro A, Varghese R, Arasu MV, Agastian P, Al-Dhabi NA, Ilavenil S, Choi KC (2018) In vitro investigation on probiotic, anti-Candida, and antibiofilm properties of *Lactobacillus pentosus* strain LAP1. Arch Oral Biol 89:99–106. 10.1016/j.archoralbio.2018.02.014 29499562 10.1016/j.archoralbio.2018.02.014

[33] Schmidt-Arras D, Rose-John S (2016) IL-6 pathway in the liver: from physiopathology to therapy. J Hepatol 64:1403–1415. 10.1016/j.jhep.2016.02.004 26867490 10.1016/j.jhep.2016.02.004

[34] Ivashkiv LB (2018) IFN*γ*: signalling, epigenetics and roles in immunity, metabolism, disease and cancer immunotherapy. Nat Rev Immunol 20:85–86. 10.1038/s41577-018-0029-z 10.1038/s41577-018-0029-z PMC6340644 29921905

[35] Terneus MV, Brown JM, Carpenter AB, Valentovic MA (2008) Comparison of S-adenosyl-L-methionine (SAMe) and N-acetylcysteine (NAC) protective effects on hepatic damage when administered after acetaminophen overdose. Toxicology 244:25–34. 10.1016/j.tox.2007.10.027 18068290 10.1016/j.tox.2007.10.027 PMC2247417

[36] Lash LH, Jones DP (1985) Distribution of oxidized and reduced forms of glutathione and cysteine in rat plasma. Arch Biochem Biophys 240:583–592. 10.1016/0003-9861(85)90065-7 4026295 10.1016/0003-9861(85)90065-7

[37] Masella R, Di Benedetto R, Varì R, Filesi C, Giovannini C (2005) Novel mechanisms of natural antioxidant compounds in biological systems: involvement of glutathione and glutathione-related enzymes. J Nutr Biochem 16:577–586. 10.1016/j.jnutbio.2005.05.013 16111877 10.1016/j.jnutbio.2005.05.013

[38] Wendel A, Cikryt P (1980) The level and half-life of glutathione in human plasma. FEBS Lett 120:209–211. 10.1016/0014-5793(80)80299-7 7439398 10.1016/0014-5793(80)80299-7

[39] Polavarapu R, Spitz DR, Sim JE, Follansbee MH, Oberley LW, Rahemtulla A, Nanji AA (1998) Increased lipid peroxidation and impaired antioxidant enzyme function is associated with pathological liver injury in experimental alcoholic liver disease in rats fed diets high in corn oil and fish oil. Hepatology 27:1317–1323. 10.1002/hep.510270518 9581686 10.1002/hep.510270518

[40] Yuan R, Tao X, Liang S, Pan Y, He L, Sun J, Wenbo J, Li X, Chen J, Wang C (2018) Protective effect of acidic polysaccharide from *Schisandra chinensis* on acute ethanol-induced liver injury through reducing CYP2E1-dependent oxidative stress. Biomed Pharmacother 99:537–542. 10.1016/j.biopha.2018.01.079 29902864 10.1016/j.biopha.2018.01.079

[41] Pastore A, Federici G, Bertini E, Piemonte F (2003) Analysis of glutathione: implication in redox and detoxification. Clin Chim Acta 333:19–39. 10.1016/s0009-8981(03)00200-6 12809732 10.1016/s0009-8981(03)00200-6

[42] Chuang CH, Tsai CC, Lin ES, Huang CS, Lin YY, Lan CC, Huang CC (2016) Heat-killed *Lactobacillus salivarius* and *Lactobacillus johnsonii* reduce liver injury induced by alcohol in vitro and in vivo. Molecules 21:1456. 10.3390/molecules21111456 27809254 10.3390/molecules21111456 PMC6274176

[43] Osna NA, Donohue TM Jr, Kharbanda KK (2017) Alcoholic liver disease: pathogenesis and current management. Alcohol Res 38:147–161 28988570 10.35946/arcr.v38.2.01 PMC5513682

[44] Barone R, Rappa F, Macaluso F, Caruso Bavisotto C, Sangiorgi C, Di Paola G, Tomasello G, Di Felice V, Marcianò V, Farina F, Zummo G, Conway de Macario E, Macario AJ, Cocchi M, Cappello F, Marino Gammazza A (2016) Alcoholic liver disease: a mouse model reveals protection by *Lactobacillus fermentum*. Clin Transl Gastroenterol 7:e138. 10.1038/ctg.2015.66 26795070 10.1038/ctg.2015.66 PMC4737872

[45] Tsai YS, Lin SW, Chen YL, Chen CC (2020) Effect of probiotics *Lactobacillus paracasei* GKS6, *L. plantarum* GKM3, and *L. rhamnosus* GKLC1 on alleviating alcohol-induced alcoholic liver disease in a mouse model. Nutr Res Pract 14:299–308. 10.4162/nrp.2020.14.4.299 32765811 10.4162/nrp.2020.14.4.299 PMC7390740

[46] Wang R, Tang R, Li B, Ma X, Schnabl B, Tilg H (2021) Gut microbiome, liver immunology, and liver diseases. Cell Mol Immunol 18:4–17. 10.1038/s41423-020-00592-6 33318628 10.1038/s41423-020-00592-6 PMC7852541

[47] Llopis M, Cassard AM, Wrzosek L, Boschat L, Bruneau A, Ferrere G, Puchois V, Martin JC, Lepage P, Le Roy T, Lefèvre L, Langelier B, Cailleux F, González-Castro AM, Rabot S, Gaudin F, Agostini H, Prévot S, Berrebi D, Ciocan D, Jousse C, Naveau S, Gérard P, Perlemuter G (2016) Intestinal microbiota contributes to individual susceptibility to alcoholic liver disease. Gut 65:830–839. 10.1136/gutjnl-2015-310585 26642859 10.1136/gutjnl-2015-310585

[48] Capurso G, Lahner E (2017) The interaction between smoking, alcohol and the gut microbiome. Best Pract Res Clin Gastroenterol 31:579–588. 10.1016/j.bpg.2017.10.006 29195678 10.1016/j.bpg.2017.10.006

[49] Ozer J, Ratner M, Shaw M, Bailey W, Schomaker S (2008) The current state of serum biomarkers of hepatotoxicity. Toxicology 245:194–205. 10.1016/j.tox.2007.11.021 18291570 10.1016/j.tox.2007.11.021

[50] Zoppini G, Cacciatori V, Negri C, Stoico V, Lippi G, Targher G, Bonora E (2016) The aspartate aminotransferase-to-alanine aminotransferase ratio predicts all-cause and cardiovascular mortality in patients with type 2 diabetes. Medicine (Baltimore) 95:e4821. 10.1097/MD.0000000000004821 27787357 10.1097/MD.0000000000004821 PMC5089086

[51] Farahmand SK, Samini F, Samini M, Samarghandian S (2013) Safranal ameliorates antioxidant enzymes and suppresses lipid peroxidation and nitric oxide formation in aged male rat liver. Biogerontology 14:63–71. 10.1007/s10522-012-9409-0 23179288 10.1007/s10522-012-9409-0

[52] Kirpich IA, Solovieva NV, Leikhter SN, Shidakova NA, Lebedeva OV, Sidorov PI, Bazhukova TA, Soloviev AG, Barve SS, McClain CJ, Cave M (2008) Probiotics restore bowel flora and improve liver enzymes in human alcohol-induced liver injury: a pilot study. Alcohol 42:675–682. 10.1016/j.alcohol.2008.08.006 19038698 10.1016/j.alcohol.2008.08.006 PMC2630703

[53] Alves-Bezerra M, Cohen DE (2017) Triglyceride metabolism in the liver. Compr Physiol 8:1–8. 10.1002/cphy.c170012 29357123 10.1002/cphy.c170012 PMC6376873

[54] Brody H (2017) Fatty liver disease. Nature 551:7681 10.1038/d41586-017-06927-0 29168811

[55] Cohen SM (2016) Alcoholic liver disease. Clin Liver Dis. 10.1016/j.cld.2016.05.001 27373620 10.1016/j.cld.2016.05.001

